# The Pitfalls of Genomic Data Diversity

**DOI:** 10.1002/hast.1511

**Published:** 2023-11-14

**Authors:** Anna Jabloner, Alexis Walker

**Keywords:** genomics, race and racism, equity in health care, finances, bioethics

## Abstract

*Biomedical research recruitment today focuses on including participants representative of global genetic variation—rightfully so. But ethnographic attention to practices of inclusion highlights how this agenda often transforms into “predatory inclusion,” simplistic pushes to get Black and brown people into genomic databases. As anthropologists of medicine, we argue that the question of how to get from diverse data to concrete benefit for people who are marginalized cannot be presumed to work itself out as a byproduct of diverse datasets. To actualize the equitable translation of genomics, practitioners need to place the impacts of ancestral genetic difference in the scope of much more impactful social determinants. For this to happen, multidisciplinary expertise needs to be leveraged, and current, structurally unequal health care systems ultimately need to transform. As modest steps toward this goal, new models for benefit‐sharing must be developed and implemented to mitigate existing inequality between data donors and the entities profiting from that data*.

## Essay

It has become common for health researchers and practitioners to express concern that cutting‐edge technologies are not well tailored for minoritized people who, despite long being the subjects of medical experimentation, are underrepresented in today's large‐scale health data banks and biobanks.^1^ However, there is a crucial blind spot in today's proliferating efforts to address this issue by diversifying health datasets: the thinking behind such efforts has generally failed to work out how to get, in practice, from diverse data to concrete benefit for the most marginalized. These efforts rely on the flawed idea that benefit for underserved populations will flow directly from more diverse datasets and associated biomechanistic research tailored to “minority populations.”

Approaches to bias are in urgent need of revision across the biomedical sciences; the field of genomics provides a significant example. Our research on the uses of racial and ancestry categories in genomics suggests that, in practice, in the process of communication and implementation, calls for genomic data diversity often transform into simplistic pushes to get more Black and brown people into research and databases, such as the U.S. National Institutes of Health's (NIH's) All of Us Program.^2^ The mantra of “getting more people of color” into genomic databases reproduces the popular belief that race is genetic; social scientists have firmly demonstrated that treating race this way can impede efforts to address social drivers of health.^3^ Crucially, the focus on diversifying datasets exemplifies what Black studies scholar Keeanga‐Yamahtta Taylor calls “predatory inclusion,”^4^ a type of inclusion that works to the advantage of those who already hold power and not necessarily to the benefit of those who are being recruited. From our perspective as medical anthropologists who study health justice, efforts to diversify health research need to go hand in hand with more robust benefits for data donors (including financial benefits) and much deeper education of the genomics workforce around social dynamics of health. To move the needle on health equity, one cannot simply assume that more diverse datasets will automatically lead to better health care for the underserved. Researchers and funders must develop research programs that fill in the gaps of this process; key here are the financial relationships surrounding health research.

Health datasets have important financial implications: for example, they are an essential resource in developing lucrative new health technologies, U.S. hospitals are regularly selling access to patient data, and genomic datasets have driven equity investment in companies on the order of hundreds of millions of dollars.^5^ While researchers in both industry and academic settings reap financial benefit from their work, for people of non‐European ancestry—whose data is in extremely high demand in efforts to diversify health research—there is only a promise of better health care in the future, with no concrete plan for how that benefit will be realized.^6^ In the context of biomedical and health research across the globe, where our empirical research has shown academic and economic considerations to be closely entangled,^7^ good diversity numbers are now also considered a financial necessity for university‐ and industry‐based research. But the path to equity cannot simply be presumed to work itself out as a byproduct of more diverse datasets.

Genomics researchers and investors (from researchers at public agencies to venture capitalists) consistently cite health equity as a central pillar of today's genomics endeavors. This approach to garnering public support compounds the moral obligation of researchers and funders to take real action in addressing those inequalities and their primary drivers—including current forms of capitalist biomedicine. Structurally entrenched racism has supported a long and ongoing history in which researchers and other powerful parties have used people of color as physical resources for social improvement (both in health research and more broadly) while excluding those same people from the financial benefits of such improvement—capitalizing on inequality.^8^

Recognizing the ways that racial and capitalist dynamics continue to shape the biosciences is crucial to any move toward justice in this arena. Indigenous genomic scholars Krystal Tsosie and colleagues, for example, outline a continuous cycle in which research harms to Indigenous Americans lead to disengagement from research participation, followed by recruitment efforts in which “indigenous people are told they will miss precision health benefits if they do not engage.”^9^ But in the face of this threatening language from health researchers and policy‐makers, Tsosie and colleagues note the validity of refusing to participate in research that is rooted in the current biocapitalist framework that makes data a commodity while doing little to help the inequities Indigenous Americans face.

Amidst the wide range of efforts necessary to substantially alleviate inequality in the United States and more broadly, we understand models of financial benefit sharing as one complimentary element. In genomics, some parties are pursuing new models of sharing the “benefits” of research, in an attempt to address economic inequality between data donors and the entities profiting from that data. For instance, some companies have begun offering data donors a percentage of research and revenue streams or shares in their company. Ethicists Eman Ahmed and Mahsa Shabani outline the details of emerging “DNA marketplaces” that are intended to give more control to, or share financial benefits with, data donors.^10^ These systems are only just emerging; ideas about what counts as benefit are in flux. Examining benefit metrics in practice across health research and policy is key to elucidating underlying assumptions about how inequality can be mitigated meaningfully and to better designing these efforts to function.^11^

To be sure, these benefit‐sharing approaches cannot fully address the massive redistributions of wealth necessary to start addressing histories of structural discrimination.^12^ We acknowledge concerns about the further commoditization of human life and about undue influence to participate in research, as Ahmed and Shabani outline. However, we consider more robust financial benefit sharing (be that individual or group focused) to be a modest step toward equalizing social positions, offering concrete reward for underserved people who are recruited for biomedical research, in addition to being a more honest engagement in the context of contemporary capitalist biomedicine. Until massive social welfare redistributions allow this country to really begin addressing health inequities, efforts toward monetary compensation for data donors are a realistic step toward recognizing the value that donors offer, especially those of non‐European ancestry. Underrepresented data donors getting health care in exchange for data, with genomic companies paying for their plans, would also be one such approach. Regardless of form, resources for recruiting people of non‐European ancestry into genomics and biomedical research need to be matched by resources to *realize* benefits for those very groups.

Beyond these financial benefits, a commitment to realizing health benefits for ethnic and racial minorities participating in research requires more robust education of the genomics workforce regarding the social dynamics of race in medicine. In our research, we have been dismayed to witness leaders in genomics companies treating race as only a biological category (an understanding of race that has been strongly refuted by the American Society of Human Genetics, among other consensus bodies) and speaking of genomic science as existing outside the sphere of economics. Addressing inequities here requires a workforce that understands how to accurately place the impacts of ancestral genetic difference in the scope of the much more impactful social determinants of health, making education a crucial priority for companies and research institutions. At a minimum, those working to improve the diversity of genomic datasets must be able to understand when research on ancestry categories is merited and how to use more‐accurate frameworks focused on narrow and precise ancestry categories in health and biomedical programs.^13^ In addition, a basic understanding of the sociohistory of genomics within and as part of economic processes, as well as of the history of racism within and as part of economic processes, is key to developing health‐benefit metrics that are realizable within the confines of current capitalist biomedicine.

To address inequality, sustained bridging of the gulf between social and medical scientists is also essential, in particular, to bring social science insights such as those cited above into biomedical practice. Even as social scientists are being integrated into biomedical projects, such as through the NIH's Ethical, Legal, and Social Implications of Genomics program, such efforts often remain an add‐on to the “real” science, largely operating separately from the bioscience elements rather than deeply informing them.^14^ In attempts to bring social and biological issues into the same picture, the social is often examined through those biological processes and methodologies that are in the purview of genomic and biomedical sciences. But if ensuring equitable benefit, or even addressing health inequality, is really the goal, the analytic insights from the social, human, and cultural sciences—precisely those identifying capitalist biomedicine, the enduring effects of history, and the structural impacts of oppression even in the absence of direct interpersonal discrimination—are essential to designing successful interventions. Bridging the gulf between social and medical scientists would mean that the analytic contributions of the social and human sciences in understanding and overcoming inequality are not skirted in efforts to address racial health inequality and are instead brought to the core of the scientific effort.

If addressing inequality is to be more than lip service, leaders in genomics need to leverage real resources for the valuable donors of diverse data and the education of the genomics workforce. These and other powerful actors should also invest financial resources into meaningful, long‐term translation efforts between the natural and the social, human, and cultural sciences. In the meantime, while biomedical researchers may not be able to transform the health care system, they are well positioned to mobilize financial resources for the people they aim to recruit today.

## Acknowledgments

Work on this essay was funded by the National Human Genome Research Institute grant 1K99HG010499‐01. The authors gratefully acknowledge this support. The authors also wish to thank an anonymous reviewer for the productive feedback.

## Disclaimer

The conclusions contained herein are solely the responsibility of the authors and do not represent the official views of the National Institutes of Health.
